# Laparoscopic hemicolectomy with intracorporeal anastomosis for ascending colon cancer in situs inversus totalis: a case report and literature review

**DOI:** 10.3389/fonc.2026.1967046

**Published:** 2026-09-16

**Authors:** Yucheng Li, Yuanyi Rui, Bo Yi, Yangchun Zheng

**Affiliations:** 1 Colorectal and Gastrointestinal Surgery, West China Tianfu Hospital, Sichuan University, Chengdu, China; 2 Department of Colorectal Surgery, Sichuan Cancer Hospital, Medical School of University of Electronic Science and Technology of China, Chengdu, China

**Keywords:** ascending colon cancer, colorectal surgery, intracorporeal anastomosis, laparoscopic radical right hemicolectomy, situs inversus totalis (SIT)

## Abstract

Ascending colon cancer complicated by situs inversus totalis (SIT) represents an extremely rare clinical entity. This congenital anatomical variant substantially increases surgical complexity, and published clinical reports regarding this condition remain scarce. Herein, we report a patient who underwent laparoscopic radical right hemicolectomy (LRH) with intracorporeal anastomosis (IA), accompanied by an operative video. We further reviewed the available published literature to accumulate additional surgical experience for managing this rare disease.

## Introduction

SIT is an uncommon congenital anatomical malformation characterized by complete mirror-image reversal of thoracic and abdominal visceral organs (1). Its overall prevalence is approximately 0.01% (2, 3). Ascending colon cancer with situs inversus totalis (ACC-SIT) is even rarer. Owing to the mirrored displacement of visceral organs and vascular structures, conventional surgical approaches, surgeon positioning, trocar configuration, and instrument manipulation trajectories must be modified accordingly (1, 4), rendering laparoscopic procedures technically more demanding in SIT patients.

IA confers benefits including reduced postoperative pain and lower surgical-site incision-related complications. To date, only one published case has described IA performed for transverse colon cancer in the setting of SIT (5). The present case represents the first reported application of IA for ACC-SIT. LRH constitutes the primary therapeutic modality for ACC-SIT; nevertheless, standardized clinical guidelines or expert consensus specifically tailored to ACC-SIT are lacking. Therefore, we performed a systematic review of relevant literature to provide supplementary practical surgical insights for this rare pathology.

## Case presentation

A 56-year-old female patient (height: 155 cm; body weight: 71 kg; body mass index (BMI) = 29.55) was admitted on March 27, 2024, with a chief complaint of diarrhea. Computed tomography (CT) demonstrated imaging features consistent with ACC-SIT (**Figures 1A, B**). Colonoscopy identified an ascending colonic neoplasm causing luminal stenosis. Following comprehensive preoperative risk assessment and acquisition of written informed consent, the patient received LRH combined with IA.

**Figure 1:**
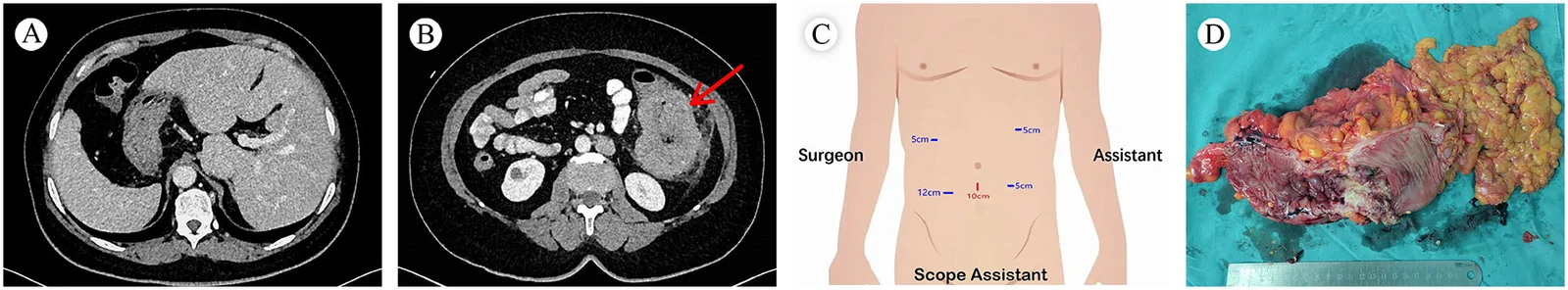
**(A)** CT findings: Visceral organ arrangement in a patient with SIT. **(B)** CT findings: Red arrow indicates the ascending colonic tumor. **(C)** Placement of abdominal trocars and positional layout of surgical staff. **(D)** Resected ascending colon surgical specimen.

After successful induction of general endotracheal anesthesia, the patient was positioned in the supine lithotomy position. The primary surgeon stood on the patient’s right side, the surgical assistant was positioned on the patient’s left side, and the laparoscopic camera assistant stood between the patient’s lower extremities. A total of five trocars were placed (**Figure 1C**). The observation trocar was inserted approximately 5 cm below the umbilicus. A 12-mm trocar for the primary surgeon’s right-hand instrumentation was placed at the McBurney’s point, and the operating port for the primary surgeon’s left-hand instrumentation was sited below the costal margin along the right mid-clavicular line. Trocars for the surgical assistant were placed symmetrically relative to the primary surgeon’s ports.

After establishment of pneumoperitoneum, thorough abdominal exploration revealed no intra-abdominal tumor seeding or hepatic metastatic lesions. The ascending colonic tumor was located within the left abdominal cavity (**Figure 2A**). Following the principle of complete mesocolic excision (CME), we performed en-bloc resection of the ascending colon together with its intact mesentery, with central vascular ligation and adequate regional lymphadenectomy. The ileocolic mesenteric bridge was incised to access the Toldt’s space. The ileocolic artery (ICA) and ileocolic vein (ICV) were ligated and divided, followed by regional lymph node dissection (**Figure 2B**). Dissection within Toldt’s space was continued. Guided by the superior mesenteric vein (SMV), the right branch of the middle colic artery (MCA-R) was identified, ligated, and transected (**Figure 2C**). Subsequently, the left branch of the middle colic artery (MCA-L) was isolated, ligated, and divided (**Figure 2D**). The Henle’s trunk was dissected on the left side of the SMV, after which the middle colic vein (MCV) was identified, ligated, and cut (**Figure 2E**). The anterior pancreatoduodenal space and pre-renal space were further developed. The retrocolic anatomical planes under situs-inversus conditions are illustrated (**Figure 2F**).

**Figure 2:**
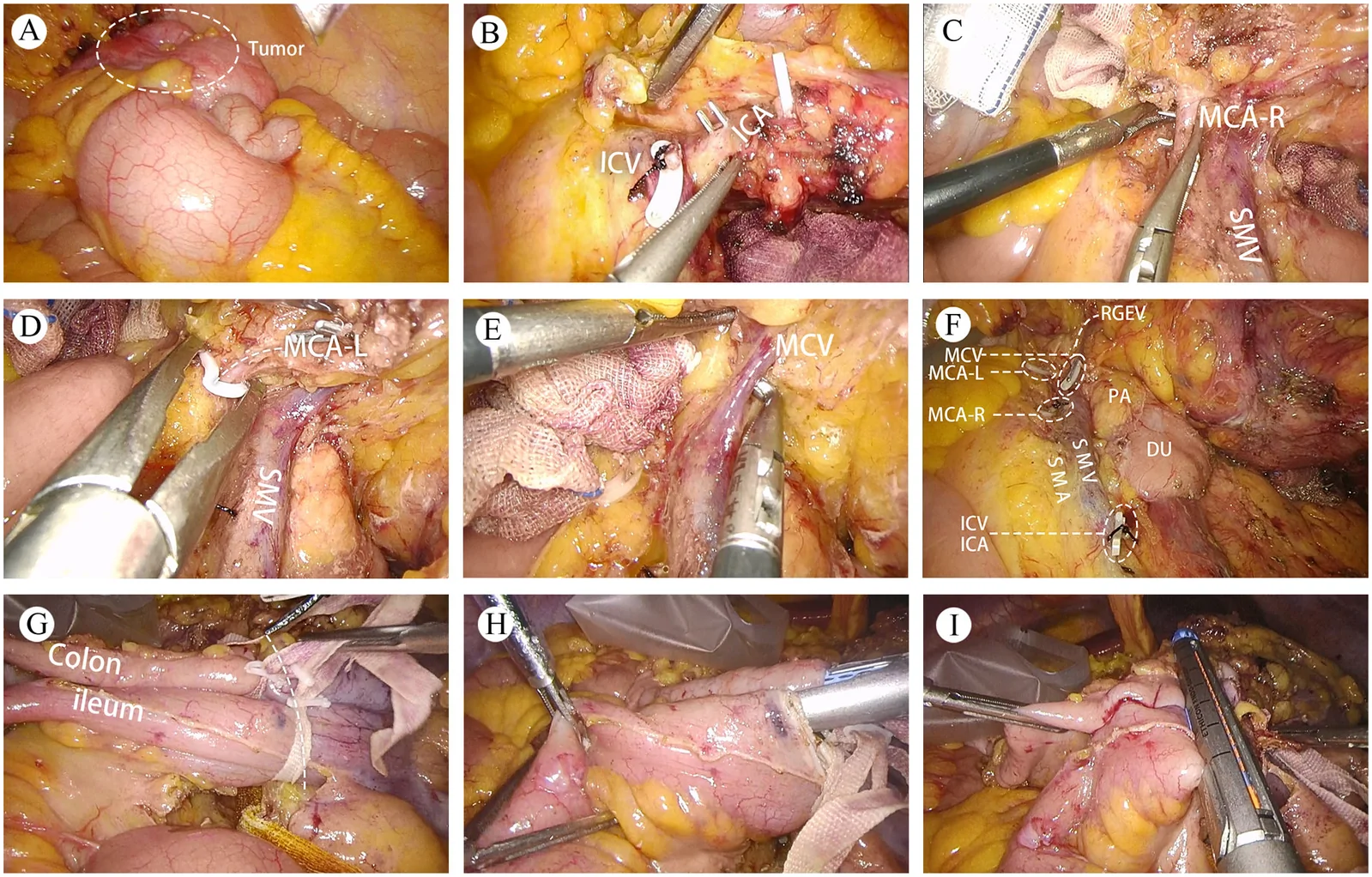
Intraoperative surgical steps. **(A)** Location of the ascending colon tumor. **(B)** Ligation and division of ICA and ICV. **(C)** Ligation of MCA-R. **(D)** Ligation of MCA-L. **(E)** Ligation of MCV. **(F)** Exposure of the retro-ascending-colon space (DU: Duodenum; PA: Pancreas; RGEV: Right Gastroepiploic Vein). **(G)** Bowel ligation at the ischemic demarcation line. **(H)** Ileocolic side-to-side anastomosis. **(I)** Closure of the common enterotomy.

The greater omentum was divided to open the omental bursa, and the gastrocolic ligament was transected. On the left abdominal side, the colonic lateral peritoneal ligament was incised; upward mobilization and division of the hepatocolic ligament enabled complete mobilization of the hepatic flexure of the colon. The transverse colon and terminal ileum were encircled and ligated at the anticipated demarcation of intestinal ischemia (**Figure 2G**). Bowel walls were incised, and side-to-side IA was completed using a laparoscopic linear stapler (**Figure 2H**). The common enterotomy orifice was closed with another laparoscopic linear cutting stapler (**Figure 2I**). Barbed suture was applied for continuous suture reinforcement of the stapled closure line. Interrupted sutures were placed at the anastomosis site to relieve anastomotic tension. The mesenteric defect was closed with continuous suturing. A limited abdominal wall incision was extended for retrieval of the surgical specimen (**Figure 1D**).

The total operative duration was 440 min, and the estimated intraoperative blood loss was 50 mL. The patient experienced no postoperative adverse events including anastomotic leakage, gastrointestinal bleeding, or postoperative ileus. She was discharged uneventfully on postoperative day 7. Postoperative histopathology confirmed ascending colon adenocarcinoma with a maximal tumor diameter of 7.5 cm; the pathological stage was pT3N0M0. None of the 30 harvested lymph nodes harbored metastatic carcinoma. The patient remained alive without evidence of tumor recurrence at 26-month postoperative follow-up.

## Discussion

Colorectal cancer (CRC) ranks as the third most prevalent malignancy worldwide (6). Nevertheless, colorectal cancer with situs inversus totalis (CRC-SIT) is exceedingly rare; ACC-SIT accounts for approximately 29% of such cases (7). Owing to limited early clinical awareness of CRC-SIT, open surgical resection predominated in earlier eras. With the popularization and technical refinement of laparoscopic surgery, its advantages of minimal invasiveness and accelerated postoperative recovery have been well-recognized (8), and laparoscopic approaches have gradually become the standard therapeutic option. However, the aberrant visceral anatomy associated with SIT increases intraoperative technical challenges (9). Given the scarcity of SIT-related cases, surgeons face a steep learning curve, which constitutes a substantial clinical challenge. Cumulative published case reports have validated the safety of laparoscopic resection for ACC-SIT (10, 11), and our clinical observation supports this conclusion.

Compared with extraluminal anastomosis, IA has more advantages in mitigating postoperative pain, lowering incision-related morbidity, and shortening hospital stay (12, 13). Moreover, IA does not compromise oncological outcomes (14). Particularly for obese patients with thick abdominal walls, extracorporeal bowel exteriorization requires longer abdominal incisions, which elevates the risk of surgical-site complications. IA permits shorter abdominal wall incisions and reduces the duration of incision exposure to enteric contents, thereby diminishing the risk of wound-related adverse events. In our case, the patient had a BMI of 29.55 and exhibited no postoperative surgical-site infection or incisional hernia. Prior robotic-surgery series have verified the safety of IA (15); however, robotic platforms remain inaccessible for many centers, where conventional laparoscopic systems constitute the mainstay of clinical practice. We performed LRH with IA for this ACC-SIT patient. No anastomotic leakage, postoperative ileus, or other major complications occurred postoperatively. No instrument-handling difficulties were encountered during the whole procedure. Accordingly, IA can be safely implemented in ACC-SIT patients without increasing operative technical demands or peri-operative complication rates. However, the learning curve of IA surgery is longer, especially for early-experience cases (16). For SIT patients undergoing IA, this creates an additional learning barrier. Horizontally flipped surgical videos from standard (non-SIT) laparoscopic right hemicolectomy can effectively simulate the mirrored anatomical features of SIT (5). This educational modality enables surgeons inexperienced with SIT-related procedures to familiarize themselves with operative workflows and may help shorten the learning curve.

Insufficient anastomotic perfusion represents another major concern during laparoscopic colectomy for SIT patients. Inadequate perfusion is a well-recognized risk factor for anastomotic leakage (17). Therefore, precise identification of the intestinal ischemic demarcation intraoperatively is mandatory, and all non-viable intestinal segments must be resected en bloc with the primary tumor. Owing to mirrored and variant mesenteric vasculature, intraoperative assessment of bowel viability relying solely on laparoscopic white-light visualization can be unreliable. Indocyanine green-fluorescence angiography (ICG-FA) enables objective real-time evaluation of intestinal perfusion, helping detect under-perfused bowel segments and tailor resection margins to reduce anastomotic leak risk. Although ICG-FA was not utilized in our case, we recommend its intraoperative use for SIT-associated colectomy when feasible, particularly in obese patients or those with severely distorted vascular anatomy. For our patient, the magnified vision provided by laparoscopy enabled us to clearly recognize the ischemic demarcation of the bowel, which ensured no postoperative anastomotic leakage occurred.

With regard to functional outcomes following side-to-side anastomosis, a systematic review compared isoperistaltic versus antiperistaltic anastomotic configurations (18).This review demonstrates overall comparable safety profiles between the two configurations, whereas isoperistaltic anastomosis may facilitate earlier recovery of gastrointestinal function. However, each approach carries inherent drawbacks. In the mirrored anatomical setting of SIT, adequate tension-free anastomosis is more challenging for the isoperistaltic configuration, whereas antiperistaltic anastomosis theoretically increases the risk of intraluminal stasis at the anastomosis. Altered bowel orientation in SIT further complicates bowel alignment before stapler firing, constituting a unique risk for laparoscopy-assisted IA. Our patient received an antiperistaltic ileocolic anastomosis and intestinal peristalsis function was successfully restored. In addition to conventional stapled intracorporeal reconstruction, scholars described a novel U-tied semi-manual anastomosis technique for totally laparoscopic colectomy, which reduces stapler cartridge consumption (19). These simplified reconstructive approaches are worthy of consideration even in SIT patients.

We summarized published cases of SIT patients receiving LRH; together with our case, a total of 16 cases were compiled (**Table 1**). Reported operative times ranged from 91 to 440 min, and maximal intraoperative blood loss was 120 mL. The number of retrieved lymph nodes varied between 11 and 82. Prolonged operative time in our case could be attributed to the patient’s obesity and the additional technical steps required for IA. All included patients achieved favorable postoperative recovery without anastomotic leakage, hemorrhage, or ileus. Overall operative duration and length of hospital stay for SIT patients were comparable to those of patients undergoing conventional colectomy for non-SIT disease (20, 21). Our collective analysis corroborates the safety profile of laparoscopic resection for SIT-associated colon cancer. Among the reviewed literature, four studies reported postoperative follow-up data spanning 6–30 months; all enrolled patients survived without tumor recurrence. Similarly, our patient showed no signs of tumor relapse or metastatic spread at the last follow-up. These observations indicate that laparoscopic resection does not increase oncologic recurrence risk for SIT-affected patients.

**Table 1:** Literature review of patients with SIT undergoing LRH.

Author	Publication year	Tumor location	Surgical modality	Pathological stage	Number of harvested lymph nodes	Operative time (min)	Intraoperative blood loss (mL)
Yushi Fujiwara (30)	2007	Ascending colon cancer	LRH	pT3N+M0	Not reported	191	60
Hye Jin Kim (31)	2011	Colon cancer of the hepatic flexure	LRH	pT3N0M0	Not reported	119	Negligible
Yasumitsu Hirano (26)	2015	Cecal cancer	LRH with single-incision	pT3N0M0	25	125	Negligible
Kazuhito Sasaki (20)	2017	Ascending colon cancer	LRH	pT1N1M0	17	109	10
Maria Labalde Martínez (21)	2017	Ascending colon cancer	LRH	pT3N0M0	60	120	<100
Seung-Seop Yeom (28)	2018	Proximal transverse colon cancer	reduced-port laparoscopic hemicolectomy	pT3N0M0	82	170	20
Yozo Suzuki (27)	2019	Cecal cancer	LRH with single-incision	pT2N1M0	19	91	Negligible
Yutaka Kojima (24)	2019	Ascending colon cancer	LRH	pT3N1M0	11	237	20
F Marchegiani (29)	2020	Proximal ascending colon cancer	LRH with Robotic	Stage IIIB	Not reported	175	20
Ji-Long Hu (4)	2022	Ascending colon cancer	LRH	pT3N0M0	22	178	50
Sakamoto J (5)	2022	Transverse colon cancer close to the hepatic flexure	Partial laparoscopic colectomy with lymphadenectomy with IA	Not reported	Not reported	257	Negligible
Junki Kato (23)	2024	Ascending colon cancer	LRH with robot-assisted	pT1N0M0	Not reported	218	5
Martin Infante Altamirano (15)	2025	Ascending colon cancer	LRH with robot-assisted	pTisN0M0	18	Not reported	5
Bowen Su (11)	2025	Cecal cancer	LRH	pT3N0M0	Not reported	180	120
Amro Mureb (32)	2026	Ascending colon cancer	LRH	pT3N0Mx	39	210	100
Present study (This study)	2026	Ascending colon cancer	LRH with IA	pT3N0M0	30	440	50

Patients with SIT universally present with vascular anatomical variations. Preoperative vascular assessment carries critical clinical significance for SIT patients to formulate individualized operative strategies and guarantee peri-operative safety (22). Three-dimensional CT and high-resolution computed-tomographic angiography (CTA) represent core preoperative diagnostic modalities (23). In SIT patients, the superior mesenteric artery (SMA) is frequently located on the left side of the abdominal cavity; the right colic artery (RCA) may be absent, while the ICA lies to the left of the SMV (11). Successful laparoscopic surgery for SIT hinges on adequate management of anomalous vessels. Given the left-sided position of the ascending colon in SIT, the surgeon typically adopts a right-sided operative approach, using the SMV as the key landmark and performing dissection along its left margin. Constant anatomical landmarks including the Treitz ligament, SMV, and cecum facilitate intraoperative vascular identification. Operative workflow commonly prioritizes exposure of the SMV followed by ligation of ileocolic vessels (24). In addition, CME is equally important for patients with colon cancer. CME principles require standard complete mesocolic excision with central vascular ligation, which improves long-term oncological outcomes without increasing peri-operative morbidity (25). Even for rare SIT-associated ascending colon cancer, CME principles should still be strictly followed despite the mirrored and variant visceral-vascular anatomy.

Appropriate surgeon positioning and trocar layout lay the foundation for successful surgery (11). Most reports recommend positioning the primary surgeon on the patient’s right side to facilitate vascular dissection; alternatively, some authors advocate placing the primary surgeon between the patient’s legs (4). A five-trocar configuration is adopted in most published cases. Minor variations in trocar placement exist across reports, and port sites should be adjusted according to individual surgeon preferences. Two single-incision laparoscopic surgery (SILS) cases have been documented, utilizing periumbilical access (26, 27). Nevertheless, SILS is technically demanding, and instrument clashing constitutes an inherent drawback; surgeons must possess sophisticated SILS proficiency to guarantee operative safety (26). One report described reduced-port laparoscopic hemicolectomy: a 4-cm periumbilical incision accommodated a single-port device, with an additional trocar placed 10 cm below the umbilicus. Reduced-port laparoscopy offers another safe and feasible alternative, promoting postoperative recovery and alleviating instrument crowding (28).

Robotic-assisted surgery also constitutes a safe therapeutic option for SIT patients. To date, only three robotic-surgery cases have been published (12, 21, 29). None of these three patients suffered postoperative complications. All authors emphasized the necessity of thorough preoperative radiological evaluation of vascular variants to mitigate intraoperative hemorrhage risk.

## Conclusions

Laparoscopic resection represents a safe therapeutic modality for ACC-SIT. Concurrent performance of IA does not increase operative complexity or peri-operative complication risks.

## Data Availability

The original contributions presented in the study are included in the article/**Supplementary Material**. Further inquiries can be directed to the corresponding author.
